# Bacteria isolated from Bengal cat (*Felis catus* × *Prionailurus bengalensis*) anal sac secretions produce volatile compounds potentially associated with animal signaling

**DOI:** 10.1371/journal.pone.0216846

**Published:** 2019-09-13

**Authors:** Mei S. Yamaguchi, Holly H. Ganz, Adrienne W. Cho, Thant H. Zaw, Guillaume Jospin, Mitchell M. McCartney, Cristina E. Davis, Jonathan A. Eisen, David A. Coil

**Affiliations:** 1 Department of Mechanical and Aerospace Engineering, University of California, Davis, California, United States of America; 2 Genome Center, University of California, Davis, California, United States of America; 3 Department of Evolution and Ecology, University of California, Davis, California, United States of America; 4 Department of Medical Microbiology and Immunology, University of California, Davis, Davis, California, United States of America; Universidad Politecnica de Cartagena, SPAIN

## Abstract

In social animals, scent secretions and marking behaviors play critical roles in communication, including intraspecific signals, such as identifying individuals and group membership, as well as interspecific signaling. Anal sacs are an important odor producing organ found across the carnivorans (species in the mammalian Order Carnivora). Secretions from the anal sac may be used as chemical signals by animals for behaviors ranging from defense to species recognition to signaling reproductive status. In addition, a recent study suggests that domestic cats utilize short-chain free fatty acids in anal sac secretions for individual recognition. The fermentation hypothesis is the idea that symbiotic microorganisms living in association with animals contribute to odor profiles used in chemical communication and that variation in these chemical signals reflects variation in the microbial community. Here we examine the fermentation hypothesis by characterizing volatile organic compounds (VOC) and bacteria isolated from anal sac secretions collected from a male Bengal cat (*Felis catus* × *Prionailurus bengalensis*), a cross between the domestic cat and the leopard cat. Both left and right anal sacs of a male Bengal cat were manually expressed (emptied) and collected. Half of the material was used to culture bacteria or to extract bacterial DNA and the other half was used for VOC analysis. DNA was extracted from the anal sac secretions and used for a 16S rRNA gene PCR amplification and sequencing based characterization of the microbial community. Additionally, some of the material was plated out in order to isolate bacterial colonies. Three taxa (*Bacteroides fragilis*, *Tessaracoccus*, and *Finegoldia magna*) were relatively abundant in the 16S rRNA gene sequence data and also isolated by culturing. Using Solid Phase Microextraction (SPME) gas chromatography-mass spectrometry (GC-MS), we tentatively identified 52 compounds from the Bengal cat anal sac secretions and 67 compounds from cultures of the three bacterial isolates chosen for further analysis. Among 67 compounds tentatively identified from bacterial isolates, 51 were also found in the anal sac secretion. We show that the bacterial community in the anal sac consists primarily of only a few abundant taxa and that isolates of these taxa produce numerous volatiles that are found in the combined anal sac volatile profile. Several of these volatiles are found in anal sac secretions from other carnivorans, and are also associated with known bacterial biosynthesis pathways. This is consistent with the fermentation hypothesis and the possibility that the anal sac is maintained at least in part to house bacteria that produce volatiles for the host.

## Introduction

Social animals use olfactory as well as acoustic and visual signals to distinguish group members from those that are not members of the group. Because odors can be shared within a group, olfactory cues are thought to provide honest and reliable signals for group membership. Scent secretions and marking behaviors play critical roles in animal communication, including intraspecific signals, such as identifying individuals and group membership, as well as interspecific signaling. Anal sacs are an odor producing organ common to many mammals, including members of the Order Carnivora (carnivorans) [1,2]. In carnivorans, anal sacs are two small structures found on each side of the anus [3], located between the internal and external sphincter muscles. The interior walls of each sac are lined with sebaceous and apocrine glands, and the anal sac secretes a foul smelling, oily substance that ranges in color from yellow to brown [4]. Anal sac secretions are used for defense by the hooded skunk (*Mephitis macroura*) [5] and the honey badger (*Mellivora capensis*) [6], territory marking by the spotted hyena (*Crocuta crocuta*) [7] and the wolf (*Canis lupus*) [8,9], individual identification by the domestic ferret (*Mustela putorius furo*) [10,11], the small Indian mongoose (*Herpestes auropunctatus*) [12], the giant panda (*Ailuropoda melanoleuca*) [13], the striped hyena (*Hyaena hyaena*) [14] and the spotted hyena (*C*. *crocuta*) [15] and sex recognition by the brown bear (*Ursus arctos*) [16], the giant panda (*A*. *melanoleuca*) [17], and Siberian weasels (*Mustela sibirica*) and steppe polecats (*M*. *eversmanni*) [11,18]. In domestic cats (*Felis catus*) and meerkats (*Suricata suricatta*) [19], anal sac secretions are used for territorial marking, and such secretions may have information about sex, reproductive state, and recognition of individuals [1,20]. Further, these chemical signals are species specific and chemical signals from scent glands in the Felidae were found to retain a phylogenetic signal [21].

The chemical composition of anal sac secretions has been analyzed in a number of animals in the Carnivora. Studies in the cheetah (*Acinonyx jubatus*), the red fox (*Vulpes vulpes*), the domestic dog (*Canis familiaris*), the coyote (*Canis latrans*), the gray wolf (*C*. *lupus*), lion (*Panthera leo*), and the small Indian mongoose (*H*. *auropunctatus*) have identified volatile short-chain free fatty acids, such as acetic acid, propanoic acid, and butanoic acid as being partially responsible for the odors [22–28]. The nature of these constituents led to the suggestion that they may be metabolites produced by bacteria in the sac from available substrates [22].

The fermentation hypothesis posits that bacteria metabolize secretions and produce volatile organic compounds, such as hydrocarbons, fatty acids, wax esters, and sulfur compounds [15,16,29] that are used in communication by the host [30,31]. Evidence in support of this hypothesis links bacterial action to specific, olfactory-mediated host behavior or to the production of certain odorants. For example, researchers have shown that trimethylamine, an odorant that plays a key role in mouse (*Mus musculus*) reproduction, requires commensal bacteria for its production [32] [33]. Researchers have also inhibited odorant production in the small Indian mongoose (*H*. *auropunctatus*) and the Eurasian hoopoe (*Upupa epops*) by treating the animals’ scent glands with antibiotics [30,34].

In this study, we investigated the fermentation hypothesis by focusing in detail on bacterial isolates collected from a single animal, available at the time of this study. We studied a Bengal cat, a hybrid between the Asian leopard cat (*P*. *bengalensis*) and the domestic cat (*F*. *catus*). We collected anal sac secretions in order to characterize their chemical profile and analyze bacterial community composition. Then we isolated and identified a set of bacteria that could be cultivated under anaerobic conditions from these samples. Volatiles produced by these isolates were identified and compared to those found in the anal sac secretions. This is the first study we know of in felines to demonstrate that bacteria isolated from anal sacs produce volatile compounds found in anal sac secretions.

## Methods

### Animal use and care

No laboratory animals were used in this research. The owner of a male Bengal cat volunteered to have the cat’s anal sacs manually expressed by a veterinarian at the Berkeley Dog and Cat Hospital in Berkeley, CA.

### Sample collection

The UC Davis Institutional Animal Care and Use Committee (IACUC) determined that no study approval was necessary for the collection of non-invasively sampled waste materials (such as anal sac secretions and feces). The client requested that the veterinarian express the anal sacs as part of a standard veterinary exam, and not for the purposes of this study.

With the owner’s consent, both left and right anal sacs were manually expressed in a male Bengal cat (*F*. *catus* × *P*. *bengalensis*) by a veterinarian at the Berkeley Dog and Cat Hospital in Berkeley, CA. Samples of anal sac secretions were collected using Puritan cotton swabs and placed in 2 mL screw cap tubes. In total seven swabs were used to collect samples: two for 16S rRNA gene PCR and sequencing, three for GC/MS analysis and two for culturing.

### DNA extraction and 16S rRNA gene sequencing and analysis

Three swabs were used for 16S rRNA gene PCR and sequencing: one from the left anal sac secretion, one from the right anal sac secretion, and one unused swab (used as a control). Each of these swabs was placed into 100% ethanol prior to DNA extraction. Genomic DNA was extracted using the MoBio PowerSoil DNA Isolation kit (MoBio, Carlsbad, CA, USA). Samples were transferred to bead tubes containing C1 solution and incubated at 65 °C for 10 minutes, followed by 3 minutes of bead beating. The remaining extraction protocol was performed as directed by the manufacturer.

DNA samples were sent to the Integrated Microbiome Resource (IMR), Centre for Comparative Genomics and Evolutionary Bioinformatics, Dalhousie University for sequencing. Bacterial diversity was characterized via PCR amplification of the 16S rRNA gene (V4-V5 region) using barcoded primers 515F and 926R [35]. PCR conditions were as follows: an initial denaturation step at 98 °C for 30 seconds, 30 cycles of 98 °C for 10 seconds, 55 °C for 30 seconds and 72 °C for 30 seconds, a final extension at 72 °C for 4 minutes 30 seconds, and a final hold at 4 °C. Prior to sequencing, the amount of input DNA per sample was normalized using a SequalPrep Normalization Plate, following the standard protocol (ThermoFisher Scientific, Wilmington, DE, USA). The final library pool was quantified using the Qubit dsDNA HS assay (Invitrogen, Carlsbad, CA). The IMR then generated 300 bp paired end sequencing reads of these PCR amplicons using v3 chemistry on an Illumina MiSeq machine.

Raw amplicon reads were demultiplexed and then processed using DADA2 v1.8, following the standard online tutorial [36]. The reads were trimmed down to 250 base pairs to remove low quality nucleotides. In addition, the quality of reads was ensured by trimming bases that did not satisfy a Q2 quality score. Reads containing Ns were discarded and we used two expected errors to filter the overall quality of the read (rather than averaging quality scores) [37]. Chimeric reads were also removed using DADA2 on a per sample basis. The remaining pairs of reads were merged into amplicon fragments and unique Amplicon Sequence Variants (ASVs) were identified. Reads that did not merge successfully were discarded. Upon completion of the DADA2 pipeline, all ASVs (n = 52) that were found in the negative control swabs were removed from the analysis, only three of these ASVs were also found in the anal sac samples. ASVs were assigned taxonomy using the DADA2 function “assignTaxonomy” and the Silva (NR v132) database [38–40]. One ASV that was assigned to “Eukaryotes” was removed. All ASVs with the same taxonomy (at the genus level) were grouped and then ranked by number of reads. No ASVs were assigned to mitochondria or chloroplast.

### Bacterial culturing and identification

Anal sac secretions from two swabs from the Bengal cat were vortexed with 1 mL Phosphate Buffer Saline (PBS). Two serial 1:10 dilutions were performed and 100 μL of each dilution was plated onto Columbia Blood Agar (CBA) and Brain Heart Infusion (BHI). Plates were incubated anaerobically in a BD GasPak EZ Container System with packets of CO_2_ generator for 5 days at 37 °C. Morphologically distinct colonies were streaked for isolation on both CBA and BHI. The 16S rRNA gene from each culture was PCR amplified and sequenced using Sanger sequencing using the 27F and 1391R primers. Taxonomy was assigned by the result of BLAST queries to the nr database at NCBI (excluding unnamed/environmental sequences), a species name was given in cases where the identity was >98% to only a single species.

### Extraction and collection of volatiles

Cultured organisms. To extract volatiles from *Bacteroides fragilis* UCD-AAL1 and *Tessaracoccus* sp. UCD-MLA, cultures were grown in 5 mL BHI anaerobically for 24 hours at 37 °C. Three biological replicates were conducted by placing 100 μL of the culture into each of three Restek (Bellefonte, PA) tubes filled with 5 mL of BHI. Two jar blanks (no media or bacteria) and two BHI media-only blanks were used as controls. The same procedure was followed for *Finegoldia magna* UCD-MLG, except that cultures were grown and incubated in BHI supplemented with 5% defibrinated sheep blood (BBHI) anaerobically for 24 hours at 37 °C.

Headspace extraction was performed with Solid Phase Microextraction (SPME) fibers (Part 57912-U, Sigma Aldrich), which had 50/30 μm thickness and DVB/CAR/PDMA coating. Two SPMEs were inserted into the headspace of each Restek tube prior to anaerobic incubation at 37 °C for 24 hours. SPME fibers were introduced by piercing the fibers through the septa insert of the lids and making sure that the fibers were exposed to volatile compounds present in the headspace without touching the media containing the bacteria. An internal standard was introduced before sampling using 1 μL of the standard solution (10 mL/L of decane-d22 in ethanol) per jar.

Anal sac samples. For the anal sac samples, single swabs (two containing anal sac fluid sample and one unused control swab) were placed individually into single septa screw cap jars that contained two SPME fibers. After a 24 hour incubation period, the SPMEs were removed. Then we performed a liquid extraction of volatiles by adding 20 mL of methanol to the jars and letting them sit for 24 hours.

### GC-MS analysis

Chromatography occurred on a 7890 GC (Agilent Technologies Inc., Santa Clara, CA) with a ZB-WAX 30 m × 250 μm capillary column, coated with a 0.25 μm film stationary phase (Part 7HG-G007-11, 100% polyethylene glycol from Phenomenex, Torrance, CA) equivalent to DB-Wax or Carbowax. Helium was used as the carrier gas at 1 ml/min in constant flow mode. The inlet was set to 260 °C and SPMEs were splitlessly desorbed during the run. The oven temperature was programmed to increase from 40 °C (held for 5 minutes) to 110 °C at a rate of 5 °C per minute, and raise to 250 °C (held for 10 min) at a rate of 40 °C per minute. A transfer line set at 250 °C led to a 5977A mass spectrometer (Agilent Technologies Inc., Santa Clara, CA) with a solvent delay of 5 minutes. The MS swept from 50 to 500 m/z. The mass spectrometer was operated in the selected scan mode. The MS source was set to 230 °C and the MS quad set to 150 °C. A standard mix of C_8_-C_20_ alkanes was analyzed to calculate the Kovats Retention Indices and to monitor control of the instruments.

Methanolic extract of cat anal secretion was analyzed by GC-MS as tert-butyldimethylsilyl (TBDMS) derivative. 2 mL out of the 20 mL was used in the analysis. Samples were placed in glass conical vials and dried, reacted with a mixture of 50 μL N-methyl-N-tert-butyldimethylsilyltrifluoroacetamide (MTBSTFA; Sigma-Aldrich Co. LLC., St Louis, MO, U.S.A.) and 50 μL acetonitrile at 60 °C for an hour. The derivatized anal sac solution was injected in duplicates into the GC-MS.

### GC-MS data analysis workflow

MassHunter Profinder B.08.00 (Agilent Technologies Inc.) was used to deconvolute, integrate and align the data. Peaks with amplitudes of less than 1000 counts were ignored. Compounds must have been present in at least 60% of replicates from one treatment to be included in statistical analyses. Peak areas were normalized to the internal standard peak area of each data file. Furthermore, a VOC from a bacterial isolate or anal swab sample must have been, on average, three times greater than the respective controls (media blanks, etc.) to be included in this analysis. Tentative compound identification was based on the combined comparing mass spectra to the NIST 2014 Library and by a comparison of the calculated matching of standard alkane retention indices (LRI) values, when available.

## Results and discussion

The 16S rRNA gene PCR sequencing and analysis of the feline anal sac showed that 98% of the reads that were placed into ASVs were assigned to six genera (Table 1). These ASVs generally represent genera that contain anaerobic members known to be associated with mammals. Representatives of the *Tessaracoccus* genus have been isolated in sediment and have also been found in the gut of mammals including the Indian rhinoceros (*Rhinoceros unicornis*) and humans [41–45]. *Bacteroides* is a genus of bacteria also often associated with mammals [46,47]. *Anaerococcus*, *Peptoniphilus* and *Finegoldia* are all Gram Positive Anaerobic Cocci (GPAC), formerly part of the *Peptostreptophilus* genus, and are found in mammalian guts and urinary tracts [48–50]. *Peptostreptococcus* is another mammalian-associated GPAC, with around 15 species in the genus. At least one member of the group has been found as an obligate anaerobic bacterium in cats and dogs [51,52].

**Table 1:** 16S rRNA gene PCR and sequencing based survey of the Bengal cat anal sacs. Table shows the number of reads mapping to Amplicon Sequence Variants (ASVs), summed by taxonomy at the genus level (e.g. everything with the same genus was collapsed into a single column). Not shown are any reads that were placed into ASVs for which the genera of the taxonomic assignments summed to less than 1% of the total number of sequencing reads.

Right Anal Sac		Left Anal Sac		
# of reads	%	# of reads	%	Genus
15250	83%	17840	83%	*Tessaracoccus*
1532	8%	1986	9%	*Anaerococcus*
709	4%	746	3%	*Finegoldia*
325	2%	390	2%	*Bacteroides*
182	1%	114	1%	*Peptoniphilus*
117	1%	111	1%	*Peptostreptococcus*
18115	98%	21187	98%	n/a

We cultured 25 bacterial isolates from this anal sac and found only *Tessaracoccus*, *Escherichia*, *Bacteroides*, *Finegoldia*, and *Clostridium* isolates under the conditions used (Table 2). Of the cultured bacteria, three isolates were members of genera found in high abundance in the anal sac: *Tessaracoccus*, *Bacteroides fragilis*, and *Finegoldia magna*. We therefore chose to focus our experimental efforts on these genera.

**Table 2:** Identified isolates in this study. Bolded taxa are those for which we generated volatile data. Taxonomic assignments were made by examining the results of blastn searches of 16S rRNA gene sequences that were generated via PCR amplification and Sanger sequencing.

Strain ID	Genus	Species
UCD-MLI	*Bacteroides*	*fragilis*
**UCD-AAL1**	***Bacteroides***	***fragilis***
UCD-MBD1A	*Clostridium*	*perfringens*
UCD-MBD2A	*Clostridium*	*perfringens*
UCD-MD1A	*Clostridium*	*perfringens*
UCD-MD1C	*Clostridium*	*perfringens*
UCD-MD1E	*Clostridium*	*perfringens*
UCD-MD2A	*Clostridium*	*perfringens*
UCD-MLB	*Escherichia*	*coli*
**UCD-MLG**	***Finegoldia***	***magna***
**UCD-MLA**	***Tessaracoccus***	**sp.**
UCD-MLH	*Tessaracoccus*	sp.
UCD-ACL3	*Tessaracoccus*	sp.
UCD-AD1A	*Tessaracoccus*	sp.
UCD-AD2B1	*Tessaracoccus*	sp.
UCD-AD2B2	*Tessaracoccus*	sp.
UCD-AD2B3	*Tessaracoccus*	sp.
UCD-AD3C	*Tessaracoccus*	sp.
UCD-MLE	*Tessaracoccus*	sp.
UCD-MLF	*Tessaracoccus*	sp.
UCD-MBD1B	*Tessaracoccus*	sp.
UCD-MBD1C	*Tessaracoccus*	sp.
UCD-MBD1D	*Tessaracoccus*	sp.
UCD-MBD1E	*Tessaracoccus*	sp.
UCD-MBD3A	*Tessaracoccus*	sp.
UCD-MD3B	*Tessaracoccus*	sp.

*Tessaracoccus* is a genus in the Propionibacteriaceae family of the phylum Actinobacteria. The characterized species in this genus are facultatively anaerobic and Gram-positive bacteria. Representatives from this genus have been isolated from a wide variety of environments, including feces from the Indian rhinoceros [44], the gut of humans [43], metalworking fluid [53], marine sediment [41], and activated sludge biomass [54]. For our work on *Tessaracoccus*, we focused on a single isolate, *Tessaracoccus* sp. UCD-MLA. This full-length 16S rRNA gene sequence was 97% identical to 16S rRNA gene sequences from multiple members of the genus and we could not assign a species ID with confidence [55].

*Bacteroides fragilis* is the type species of the genus *Bacteroides* in the Bacteroidaceae family (phylum Bacteroidetes) and is an obligate anaerobe [56]. It has previously been found in numerous places, including the oral cavity of the domestic cat [57], as well as the gut microbiome of the domestic dog [58]. The 16S rRNA gene of our chosen isolate, *Bacteroides fragilis* UCD-AAL1, was 99% identical to 16S rRNA gene sequences from representatives of this species at NCBI.

The only species in the genus of *Finegoldia* (Peptoniphilaceae family within the Firmicutes phylum), *Finegoldia magna*, is an obligately anaerobic Gram-positive coccus that is part of the normal flora of the human gastrointestinal and genitourinary tracts [49] and has been previously found in cats [59] as well as dogs [60]. The 16S rRNA gene from our single isolate, *Finegoldia magna* UCD-MLG, was 99% identical to 16S rRNA gene sequences from representatives of this species at NCBI.

### Anal sac secretion constituents and bacteria isolates headspace SPME

We tentatively identified 127 compounds from the domestic cat anal gland secretion. Out of 127 tentatively identified compounds, 89 compounds were found in liquid extraction of anal secretion after TBDMS derivatization (S1 Table), and 52 compounds were measured by SPME-GC-MS in the anal sac secretion. These compounds were tentatively identified on the basis of the precise interpretation of its accurate mass spectra, MS fragmentation, and Kovats index information. These VOC metabolites were identified in the following compound chemical classes: heterocyclic compounds (12%), alcohols (16%), fatty acids (17%), ketones (11%), aromatic carbons (13%), amines (9%), aldehydes (7%), esters (6%).

A total of 67 unique compounds were tentatively identified from the SPME analysis of the three bacterial isolates, with some compounds being found in more than one isolate (19 compounds from *B*. *fragilis* UCD-AAL1, 44 compounds from *Tessaracoccus* sp. UCD-MLA, and 23 compounds from *F*. *magna* UCD-MLG) (Table 3). Among these 67 compounds, 52 compounds were also found in the anal sac secretion. 11 compounds (octan-1-ol, 1-(H)-indole, nonanoic acid, pentadecanoic acid, toluene, trans-2-pentenoic acid, non-2-enal, tetradecanal, n-hexadecanoic acid, octadecanoic acid, and (Z)-docos-13-enoic acid) found in the anal sac secretion have also been reported in other mammalian anal sac secretions [10,13,14,17,19,20,26,27]. Octan-1-ol and 1-(H)-indole were found in the anal sac secretion and in all three bacterial isolates. Octan-1-ol is a compound that produces a pungent odor and was previously reported in *C*. *lupus* anal sac secretion [26]. 1-(H)-indole is known to be widely distributed in the natural environment and can be produced by a variety of bacteria that have a strong fecal odor. 1-(H)-indole has also been found in various mammalian anal secretions, such as *V*. *vulpes* [61], *F*. *catus* [20], *A*. *melanoleuca* [13], *C*. *lupus* [26], and *M*. *putorius furo* [10]. Toluene is an aromatic compound found naturally in petroleum and coal, and is a major component of gasoline [62]. Raymer *et*. *al*. reported toluene found in *C*. *lupus* [26]. Although anaerobic bacterial toluene degradation is a well-known pathway [63], toluene biosynthesis is less common in bacteria. It is possible that our tentatively identified result could be a different compound but is likely to contain the alkyl benzene structure. Non-2-enal, and tetradecanal are chain aldehyde compounds. Non-2-enal was previously found in *C*. *lupus* [26] and tetradecanal was found in both *S*. *suricatta* [19,27], and *A*. *melanoleuca* [17] anal sac secretions. It is likely these aldehydes are formed by bacterial oxidation of a ubiquitous fatty acid such as oleic acid [64]. Out of 52 compounds, six fatty acids were tentatively identified from the anal sac secretion here; nonanoic acid, pentadecanoic acid, trans-2-pentenoic acid, n-hexadecanoic acid, octadecanoic acid, and (Z)-docos-13-enoic acid. Fatty acids are one of the most common compound groups known to be present in various animal anal sac secretions [13,14,16,17,19,27] and all of the fatty acids found in the anal sac secretion have been previously reported in other mammalian anal sac secretions. Nonanoic acid is a nine carbon fatty acid known to have unpleasant rancid odor, and previously found in *S*. *suricatta* anal sac secretions [19]. Pentadecanoic acid was found in *P*. *leo* [27], *S*. *suricatta* [19], and *A*. *melanoleuca* [13,17] anal sac secretions. Trans-2-pentenoic acid was found in *H*. *hyaena* [14] anal sac secretions. N-hexadecanoic acid was found in *P*. *leo* [27], *S*. *suricatta* [19], *A*. *melanoleuca* [17] anal sac secretions. (Z)-docos-13-enoic acid was found in *A*. *melanoleuca* [13,17] anal sac secretions. Fatty acids are a very common group biosynthesized by variety of organisms. The biogenesis typically starts with acetyl CoA, which is extended with malonate units to ultimately assemble fatty acids containing an even number of carbons [64]. All six fatty acids found in the anal sac secretion were also found in the *Tessaracoccus* sp. UCD-MLA culture. It is possible that the highly abundant (in the sample here) *Tessaracoccus* is largely contributing in the production of fatty acid compounds in this anal sac.

**Table 3:** Tentatively identified compounds (compound name and formula listed) from cultures and anal sac. The presence of the compound is indicated by a ‘+’ in the column corresponding to each sample: Bengal cat anal sac secretion chemical profile (“Anal sac”) and headspace solid phase microextraction (SPME) for cultures of *B*. *fragilis* UCD-AAL1 (“*Bf*”), *Tessaracoccus* sp. UCD-MLA (“*Tess*”), and *F*. *magna* UCD-MLG (“*Fm*”)).

				Bacterial isolates		
Compound name	Formula	Reference	Anal sac	*Bf*	*Tess*	*Fm*
cyclohexanone	C6H10O		+	+	+	+
dimethyl trisulfide	C2H6S3		+	+	+	+
octan-1-ol	C8H18O	Wolf [8]	+	+	+	+
decan-1-ol	C10H22O		+	+	+	+
1-(H)-indole	C8H7N	Red fox [27]; Domestic cat [20]; Giant panda [13]; Wolf [8]; Ferret [10]	+	+	+	+
dimethyl disulfide	C2H6S2		+		+	+
m-xylene	C8H10		+		+	+
2,5-dimethylpyrazine	C6H8N2		+		+	+
phenylmethanol	C7H8O		+		+	+
nonanoic acid	C9H18O2	Meerkats [19]	+	+	+	
pentadecanoic acid	C15H30O2	Lion [27]; Meerkats [19]; Giant panda [13,17]	+		+	+
trichloromethane	CHCl3			+	+	+
toluene	C7H8	Wolf [8]	+		+	
2-aminocyanoacetamide	C3H5N3O		+		+	
butan-1-ol	C4H10O		+		+	
trans-2-pentenoic acid	C5H8O2	Striped hyena [14]	+		+	
Pyrazine	C4H4N2		+		+	
azetidine	C3H7N		+	+		
2-Methyl-2-propanyl acrylate	C7H12O2		+	+		
ethenylbenzene	C8H8		+		+	
2-methylpyrazine	C5H6N2		+		+	
2-methylcyclopentanone	C6H10O		+			+
methyl 3-amino-2-methylpropanoate	C5H11NO2		+		+	
2-ethylpyrazine	C6H8N2		+		+	
1-isopropoxy-1-propene	C6H12O		+			+
2-ethyl-5-methylpyrazine	C7H8N2		+		+	
2-ethylhexyl formate	C9H18O2		+		+	
2-Isobutyl-3,6-dimethyl-pyrazine	C10H16N2		+		+	
Unknown			+			+
N-methyl-1-(methylthio)-2-nitroethenamine	C4H8N2O2S		+		+	
1-(1,3-thiazol-2-yl)ethanone	C5H5NOS		+		+	
1-phenylethan-1-one	C8H8O		+			+
2-hexylaziridine	C8H17N		+	+		
methyl (Z)-N-hydroxybenzenecarboximidate	C8H9NO2		+	+		
non-2-enal	C9H17NS	Wolf [8]	+		+	
2-decen-1-ol	C10H20O		+	+		
4-methyl-2,4-dihydro-3H-1,2,4-triazol-3-thion	C3H5N3S		+		+	
2-methylhexadecan-1-ol	C17H36O		+	+		
1-ethyl-5-methyltetrazole	C9H10N4		+		+	
3-methylcinnoline	C9H8N2		+		+	
tetradecanal	C14H28O	Meerkats [8,19]; Giant panda [17]	+		+	
(Z)-(N)-(2-methylpyridin-1-ium-1-yl)benzenecarboximidate	C13H12N2O		+		+	
phenyl carbamate	C7H7NO2		+		+	
4-amino-(N)-(3-morpholin-4-ylpropyl)-1,2,5-oxadiazole-3-carboxamide	C10H17N5O3		+		+	
pentadecyl acetate	C17H34O2		+		+	
3,5-di-(tert)-butylphenol	C14H22O		+		+	
2-hexyldecan-1-ol	C16H34O		+		+	
decyl decanoate	C20H40O2		+			+
(n)-pentyldecanamide	C18H36O2		+			+
n-hexadecanoic acid	C16H32O2	Lion [27]; Meerkats, [19]; Giant panda [17]	+		+	
octadecanoic acid	C18H36O2	Lion [27]; Giant panda [13,17]; Brown bear [16]	+		+	
(Z)-docos-13-enoic acid	C22H42O2	Giant panda [13,17]	+		+	

41 compounds found in the Bengal cat anal sac secretion have not been previously described in an anal sac secretion. Cyclohexanone and dimethyl trisulfide were found from the anal sac secretion and also from all three bacteria isolates. Cyclohexanone is known to be generated by the cyclohexanol degradation metabolomic pathway, which is widely used by bacteria [65]. Including dimethyl trisulfide, volatile sulfur compounds (hydrogen sulfide, methanethiol, dimethyl sulfide, dimethyl disulfide) such methionine derived volatiles are often generated by bacteria [64] and have also been reported to be produced by the *Bacteroides* family [66]. Hydrocarbons, aliphatic alcohols, and ketones are volatiles most likely formed by modification of products of the fatty acid biosynthetic pathway [64]. Alkanes and methyl ketones are often known to be produced from decarboxylation in bacteria [64]. Methyl ketone group compounds (2-methyl cyclopentanone, 1-(1,3-thiazol-2-yl)ethanone, 1-phenylethan-1-one) were tentatively identified from the cat anal sac secretion and at least one of the bacteria isolates. Another final modification process of the fatty acid metabolomic pathway is the reduction of carboxy groups to aldehydes and into aliphatic alcohols. Several aliphatic alcohols (decan-1-ol, butan-1-ol, 2-methylhexadecan-1-ol, 2-hexyldecan-1-ol) were tentatively identified from the anal sac secretion and from bacteria isolates. Butan-1-ol and its bishomologues up to C16 have been found in different combinations in several bacteria [64,67,68]. Aromatic compounds are common natural products in plants but also known to be produced by bacteria [64]. Especially among the aromatic alcohols, such phenols, 2-penylethanol is one of the most aromatic compounds produced by diverse bacteria. In this present study, aromatic alcohols, such as benzyl alcohol, and 3,5-ditert-butylphenol were tentatively identified from both anal sac and bacterial isolates. These aromatic alcohols can be assumed to be generated by the shikimate pathway which is present only in microorganisms and plants, never in animals [69].

## Conclusions

To our knowledge, this is the first study examining either the VOC profile of domestic feline anal sacs or the VOC profiles of associated bacteria. We show that these particular feline anal sacs are dominated by only a few taxa, most of which are easily culturable under anaerobic conditions. These bacteria produce the majority of the identified volatiles in the total anal sac scent profile. In total, 127 VOCs were found in anal sac secretions, 67 VOCs found in bacteria culture, and 51 compounds found in both anal sec and bacteria samples. Our preliminary identification of these volatiles is supported by the existence of known bacterial metabolic pathways for many of these compounds. Together these results are consistent with the fermentation hypothesis and suggest that further characterization of the anal sac microbial community, as well as the VOC’s produced therein, could potentially shed light on the potentially symbiotic relationship between these microbes and their host.

## Supporting information

S1 Table Tentatively identified compounds in the anal gland secretion of headspace solid phase microextraction (SPME) and liquid extraction tert-butyldimethylsilyl (TBDMS) derivative of domestic cat. Compounds were tentatively identified by calculating their Kovats Retention Index in comparison to reported literature values and by comparison of extracted mass spectra to the NIST 2014 mass spectral library.
(DOCX)
