# Supplementary material for: Bacteria isolated from Bengal cat (Felis catus × Prionailurus bengalensis) anal sac secretions produce volatile compounds potentially associated with animal signaling
Source: PLoS One. 2019 Sep 13;14(9):e0216846. doi: 10.1371/journal.pone.0216846 (PMC6743771; doi:10.1371/journal.pone.0216846)
Supplement: S1 Table — Compounds were tentatively identified by calculating their Kovats Retention Index in comparison to reported literature values and by comparison of extracted mass spectra to the NIST 2014 mass spectral library. (DOCX) [file pone.0216846.s001.docx]

| Compounds | Match % | Retention Index (RI), Calculated | Retention Index (RI), Literature | extraction method |
| --- | --- | --- | --- | --- |
| A alkane compound |  | 923 |  | SPME headspace |
| A fatty acid |  | 923 |  | SPME headspace |
| A fatty acid |  | 960 |  | SPME headspace |
| Toluene | 87.52 | 973 | 1011 | SPME headspace |
| (Methyldisulfanyl)methane | 83.11 | 1013 |  | SPME headspace |
| 1,3-xylene | 83.91 | 1092 | 1142 | SPME headspace |
| butyl propanoate | 72.45 | 1111 | 1135 | SPME headspace |
| 2-amino-2-cyanoacetamide | 81.71 | 1123 |  | SPME headspace |
| butan-1-ol | 88.53 | 1124 | 1160 | SPME headspace |
| (E)-pent-2-enoic acid | 63.5 | 1124 |  | SPME headspace |
| 2-Heptanone | 60.5 | 1153 | 1184 | SPME headspace |
| dimethyl bicyclo[2.2.1]heptane-2,3-dicarboxylate | 55.2 | 1181 |  | SPME headspace |
| Pyrazine | 88.42 | 1185 | 1180 | SPME headspace |
| Azetidine | 73.34 | 1186 |  | SPME headspace |
| A aldehyde |  | 1186 |  | SPME headspace |
| tert-butyl prop-2-enoate | 78.02 | 1186 |  | SPME headspace |
| Pentan-1-ol | 65.58 | 1188 | 1200 | SPME headspace |
| Ethenylbenzene | 91.95 | 1229 | 1254 | SPME headspace |
| A fatty acid |  | 1230 |  | SPME headspace |
| 2-methylpyrazine | 98.19 | 1237 | 1263 | SPME headspace |
| 2-Methylcyclopentanone | 73.63 | 1256 | 1220 | SPME headspace |
| methyl 3-amino-2-methylpropanoate | 61.84 | 1257 |  | SPME headspace |
| Cyclohexanone | 83.59 | 1260 | 1282 | SPME headspace |
| A fatty acid |  | 1263 |  | SPME headspace |
| 2,5-dimethylpyrazine | 96.32 | 1290 | 1316 | SPME headspace |
| 2-ethylpyrazine | 88.37 | 1302 | 1323 | SPME headspace |
| dimethyltrisulfane | 75.44 | 1348 | 1357 | SPME headspace |
| A fatty acid |  | 1361 |  | SPME headspace |
| An alkyne |  | 1361 |  | SPME headspace |
| Nonanal | 90.99 | 1368 | 1390 | SPME headspace |
| (E)-1-propan-2-yloxyprop-1-ene | 61.25 | 1368 |  | SPME headspace |
| A fatty acid |  | 1373 |  | SPME headspace |
| 2-ethyl-5-methylpyrazine | 90.83 | 1468 | 1432 | SPME headspace |
| 2-ethylhexyl formate | 81.31 | 1474 |  | SPME headspace |
| 2,5-dimethyl-3-(2-methylpropyl)pyrazine | 70.56 | 1495 | 1506 | SPME headspace |
| 7-methyl-2H-[1,2,4]triazolo[4,3-a]pyridine-3-thione | 55.7 | 1508 |  | SPME headspace |
| Octan-1-ol | 92.56 | 1545 | 1561 | SPME headspace |
| Unknown |  | 1548 |  | SPME headspace |
| (2Z)-2-pyrrolidin-2-ylideneacetonitrile | 83.21 | 1550 |  | SPME headspace |
| (2E,6E)-nona-2,6-dien-1-ol | 62.34 | 1562 |  | SPME headspace |
| A secondary amin compound |  | 1578 |  | SPME headspace |
| A chain aldehyde compound |  | 1591 |  | SPME headspace |
| N,N,2,2-tetramethylpropan-1-amine oxide | 67.44 | 1597 |  | SPME headspace |
| 1-methoxy-3,5-dimethylbenzene | 62.12 | 1619 | 1533 | SPME headspace |
| (E)-N-methyl-1-methylsulfanyl-2-nitroethenamine | 60.55 | 1619 |  | SPME headspace |
| Methyl benzoate | 91.95 | 1623 | 1615 | SPME headspace |
| 1-(1,3-thiazol-2-yl)ethanone | 61.18 | 1647 | 1650 | SPME headspace |
| 1-phenylethanone | 72.3 | 1650 | 1671 | SPME headspace |
| 2-hexylaziridine |  | 1653 |  | SPME headspace |
| methyl (Z)-N-hydroxybenzenecarboximidate | 59 | 1653 |  | SPME headspace |
| Decan-1-ol | 76.19 | 1663 | 1664 | SPME headspace |
| Non-2-enal | 58.48 | 1705 |  | SPME headspace |
| 2-Nonen-1-ol | 62.90 | 1706 | 1692 | SPME headspace |
| 4-methyl-1H-1,2,4-triazole-5-thione | 63.22 | 1751 |  | SPME headspace |
| Benzyl alcohol | 71.73 | 1763 | 1823 | SPME headspace |
| 2-methylhexadecan-1-ol | 66.65 | 1817 |  | SPME headspace |
| tetradecan-2-one | 55.56 | 1857 | 1855 | SPME headspace |
| 1-Hydroxy-5-phenyl-tetrazol; 5-phenyl-tetrazol-1-ol | 58.57 | 1887 |  | SPME headspace |
| 3-methylcinnoline | 80.63 | 1975 |  | SPME headspace |
| 1-(2-hydroxypropoxy)propan-2-ol | 60.43 | 1975 | 1817 | SPME headspace |
| A aldehyde compound |  | 1975 |  | SPME headspace |
| A Ketone compound |  | >2000 |  | SPME headspace |
| Phenyl carbamate | 74.99 | >2000 | 2006 | SPME headspace |
| decyl decanoate | 74.29 | >2000 | 2565 | SPME headspace |
| (Z)-octadec-9-enamide | 75.69 | >2000 | 3265 | SPME headspace |
| (17S)-3-methoxy-13-methyl-6,7,8,9,11,12,14,15,16,17-decahydrocyclopenta[a]phenanthren-17-ol | 79.33 | >2000 | 2760 | SPME headspace |
| 1-Pentadecanol acetate | 74.43 | >2000 | 1931 | SPME headspace |
| 2-hexyldecan-1-ol | 75.8 | >2000 | 2310 | SPME headspace |
| Tetradecanal | 57.87 | >2000 | 2102 | SPME headspace |
| 3,5-ditert-butylphenol | 68.02 | >2000 | 2310 | SPME headspace |
| (Z)-N-(2-methylpyridin-1-ium-1-yl)benzenecarboximidate | 57.36 | >2000 | 2169 | SPME headspace |
| 1-methyl-3-phenylbenzene | 88.31 | >2000 | 2436 | SPME headspace |
| N-Benzoylalanine | 65.22 | >2000 |  | SPME headspace |
| Nonanoic acid | 69.57 | >2000 | 2180 | SPME headspace |
| 1-(H)-indole | 88.85 | >2000 | 2420 | SPME headspace |
| Erucic acid | 76.55 | >2000 | 3132 | SPME headspace |
| octyl decanoate | 67.75 | >2000 | 2910 | SPME headspace |
| n-Pentyldecanamide | 86.68 | >2000 | 2819 | SPME headspace |
| Octadecanoic acid | 73.42 | >2000 | 2565 | SPME headspace |
| n-Hexadecanoic acid | 95.41 | >2000 | 2910 | SPME headspace |
| Pentadecanoic acid | 86.66 | >2000 | 2809 | SPME headspace |
| 6-octyloxan-2-one | 64.26 | >2000 | 2565 | SPME headspace |
| 4-amino-N-(3-morpholin-4-ylpropyl)-1,2,5-oxadiazole-3-carboxamide | 86.12 | >2000 |  | SPME headspace |
| A aromatic acid |  | >2000 |  | SPME headspace |
| A aromatic compound |  | >2000 |  | SPME headspace |
| A aromatic nitrogen compound |  | >2000 |  | SPME headspace |
| A fatty acid |  | >2000 |  | SPME headspace |
| A fatty acid |  | >2000 |  | SPME headspace |
| A secondary amin compound |  | >2000 |  | SPME headspace |
| A fatty acid |  | 810 |  | TBDMS liquid extraction |
| A fatty acid TBDMS derivative |  | 864 |  | TBDMS liquid extraction |
| A fatty acid |  | 870 |  | TBDMS liquid extraction |
| A fatty acid |  | 914 |  | TBDMS liquid extraction |
| Hymexazole, TBDMS derivative | 72.42 | 951 |  | TBDMS liquid extraction |
| Propane-1,2-diol, 2TBDMS derivative | 89.06 | 984 |  | TBDMS liquid extraction |
| D-2-Aminobutyric acid, 2TBDMS derivative | 97.26 | 1033 |  | TBDMS liquid extraction |
| Methylsuccinic acid, 2TBDMS derivative | 63.67 | 1043 |  | TBDMS liquid extraction |
| L-Valine, 2TBDMS derivative | 87.14 | 1045 |  | TBDMS liquid extraction |
| Urea | 85.13 | 1047 |  | TBDMS liquid extraction |
| Benzenepropanoic acid, TBDMS derivative | 70.77 | 1048 |  | TBDMS liquid extraction |
| L-Leucine, 2TBDMS derivative | 97.13 | 1055 |  | TBDMS liquid extraction |
| 2-Octanol, TBDMS derivative | 56.26 | 1073 |  | TBDMS liquid extraction |
| A fatty acid |  | 1073 |  | TBDMS liquid extraction |
| Cinnamic acid, (E)-, TBDMS derivative | 82.7 | 1081 |  | TBDMS liquid extraction |
| 2-Ethylhexanoic acid, TBDMS derivative | 57.22 | 1089 |  | TBDMS liquid extraction |
| 5-Aminovaleric acid, 2TBDMS derivative | 94.67 | 1094 |  | TBDMS liquid extraction |
| Indole-3-carboxylic acid, 2TBDMS derivative | 55.58 | 1103 |  | TBDMS liquid extraction |
| L-Phenylalanine, 2TBDMS derivative | 69.25 | 1140 |  | TBDMS liquid extraction |
| Phthalic acid, 2TBDMS derivative | 62.75 | 1154 |  | TBDMS liquid extraction |
| 2-Thiobarbituric acid,TBDMS derivative | 64 | 1166 |  | TBDMS liquid extraction |
| A fatty acid |  | 1172 |  | TBDMS liquid extraction |
| Oxalacetic acid, enol-2TBDMS derivative | 58.08 | 1183 |  | TBDMS liquid extraction |
| L-Lysine, 3TBDMS derivative | 64.9 | 1192 |  | TBDMS liquid extraction |
| 10-Heptadecenoic acid, (Z)-, TBDMS derivative | 60.4 | 1193 |  | TBDMS liquid extraction |
| L-Dihydroorotic acid, TBDMS derivative |  | 1217 |  | TBDMS liquid extraction |
| Nonadecanoic acid, TBDMS derivative | 63.24 | 1226 |  | TBDMS liquid extraction |
| 11-Eicosenoic acid, (Z)-, TBDMS derivative | 88.79 | 1243 |  | TBDMS liquid extraction |
| A fatty acid |  | 1253 |  | TBDMS liquid extraction |
| 13-Docosenoic acid, (Z)-, TBDMS derivative | 63.18 | 1280 |  | TBDMS liquid extraction |
| A fatty acid |  | 1304 |  | TBDMS liquid extraction |
| A fatty acid |  | 1334 |  | TBDMS liquid extraction |
| 17-(1,5-Dimethylhexyl)-10,13-dimethyl-2,3,4,7,8,9,10,11,12,13,14,15,16,17-tetradecahydro-1H-cyclopenta[a]phenanthren-3-ol | 97.51 | 1361 |  | TBDMS liquid extraction |
| 5-Docosyldihydrofuran-2(3H)-one | 71.42 | 1372 |  | TBDMS liquid extraction |
| 1-Heptacosanol, TBDMS derivative | 68.16 | 1407 |  | TBDMS liquid extraction |
| Cholesta-3,5-diene | 82.89 | 1407 |  | TBDMS liquid extraction |
| Hexacosanoic acid, TBDMS derivative | 85.31 | 1412 |  | TBDMS liquid extraction |
| 1-Octacosanol, TBDMS derivative | 56.45 | 1471 |  | TBDMS liquid extraction |
